# Atorvastatin before percutaneous coronary intervention: A systematic review and meta-analysis

**DOI:** 10.1371/journal.pone.0293404

**Published:** 2024-01-02

**Authors:** Mariano García-Campa, Ramiro Flores-Ramírez, Sabrina Rojo-Garza, Edgar Francisco Carrizales-Sepúlveda, Diego Regalado-Ceballos, Raúl Reyes-Araiza, Neri Álvarez-Villalobos, Rene Rodríguez-Gutiérrez, José Ramón Azpiri-López

**Affiliations:** 1 Cardiology Service, "Dr. José Eleuterio González" University Hospital of the Autonomous University of Nuevo Leon, Monterrey, Mexico; 2 Plataforma INVEST UANL-KER Unit Mayo Clinic, School of Medicine and University Hospital "Dr. José E González", Autonomous University of Nuevo Leon, Monterrey, Mexico; Baylor Scott and White, Texas A&M College of Medicine, UNITED STATES

## Abstract

Atorvastatin is widely recommended for long-term secondary prevention in STEMI patients with no contraindication. Although high-dose atorvastatin has been shown to reduce important patient outcomes such as MACE, there is still doubt that high-dose atorvastatin could have the same protective effect in patients undergoing PCI in the short and long term. We searched the following electronic databases: Scopus, Web of Science, MEDLINE, EMBASE, and Cochrane Central considering studies that enrolled adult patients with a confirmed diagnosis of STEMI or NSTEMI undergoing PCI. The intervention must have been atorvastatin alone compared to a placebo, standard care, or a different atorvastatin dose. A total of (n = 11) studies were included in the quantitative analysis. Information on (N = 5,399) patients was available; 2,654 were assigned to receive high-dose atorvastatin therapy, and 2,745 comprised the control group. High-dose atorvastatin pre-loading significantly reduced MACE at one month of follow-up (RR: 0.78; 95% CI: 0.67–0.91; p = 0.014) in both STEMI and NSTEMI. All-cause mortality was reduced in patients with STEMI (RR: 0.28; 95% CI: 0.10–0.81; p = 0.029). The quality of the body of evidence was rated overall as moderate. Patients presenting with STEMI or NSTEMI benefit from high-dose atorvastatin pre-loading before PCI by reducing MACE at 30 days. The use of high-dose atorvastatin in STEMI patients reduced all-cause mortality. The beneficial effects of atorvastatin pre-loading are limited to 30 days post-PCI.

## Introduction

Percutaneous coronary interventions (PCI) have revolutionized the management of ischemic heart disease (IHD). This change has been possible due to constant improvements in the technique but in higher regard to the modern technological advances and materials implemented for PCI. Optimal medical therapy (OMT) alone is not inferior to PCI plus OMT in treating patients with stable coronary disease, but PCI gains relevance in patients with acute coronary syndromes (ACS), especially in ST-segment elevation myocardial infarction (STEMI) and non-ST-segment elevation myocardial infarction (NSTEMI) [1, 2]. Medical therapy after PCI in STEMI and NSTEMI has been broadly studied and includes dual antiplatelet therapy (DAPT) with aspirin and P2Y_12_ inhibitors to lower-quality recommendations such as oral anticoagulants. Atorvastatin has also been studied due to its pleiotropic effects. It is widely recommended for long-term secondary prevention in STEMI patients with no contraindication [3, 4]. Although high-intensity statin therapy with atorvastatin (80 mg) has been shown to reduce important patient outcomes such as major adverse cardiovascular events (MACE) in ACS patients, there is still doubt that it could have the same protective effect in patients undergoing PCI in the short and long term [5]. The SECURE-PCI trial tried to approach this question. This study included patients with an ACS and planned PCI in the next seven days to receive atorvastatin (two loading doses). Two doses of 80 mg of atorvastatin did not reduce the rate of MACE. In patients who received PCI, there was a reduction in MACE at a 30-day follow-up visit, as well as non-PCI-related myocardial infarction. A post-hoc analysis showed a significant reduction in MACE in patients with STEMI treated with PCI and atorvastatin. In contrast, a clear reduction of MACE was not found in patients with non-ST elevated myocardial infarction treated with atorvastatin [6]. This systematic review and meta-analysis evaluated the efficacy of loading doses of atorvastatin before PCI in patients with STEMI or NSTEMI.

## Methods

### Study design

This protocol adhered to the Preferred Reporting Items for Systematic Review and Meta-Analysis (PRISMA) statement and was successfully registered in the International Prospective Register of Systematic Reviews (PROSPERO, NIHR) ID **CRD42022320136** [7]. (S1 Table).

### Eligibility criteria

Articles were included if they reported randomized controlled trials (RCTs) or quasi-RCTs comparing atorvastatin with a placebo or standard care in the management of STEMI and NSTEMI using a PCI. We considered studies that enrolled adult patients (>18 years) with a confirmed diagnosis of STEMI or NSTEMI undergoing PCI. The intervention must have been atorvastatin alone compared to placebo, standard care, or atorvastatin. The primary outcomes of interest were MACE and all-cause mortality. The secondary outcomes included target vessel revascularization, myocardial infarction (MI), angiographic no-reflow, TIMI flow grade III after PCI, and peak CK-MB. Patients needed to have at least 24 hours of follow-up after the PCI. Studies that administrated atorvastatin with another type of drug, except for the hospitals’ management protocol (e.g., antiplatelet therapy), were excluded. No restrictions were applied regarding the study setting or time; studies published in languages other than English were assessed for a proper medical translation.

### Search strategy

An experienced librarian, with the aid of a cardiologist, helped us design and conduct the search strategy. We searched the following electronic databases from their inception to September 2022: Scopus, Web of Science, MEDLINE, EMBASE, and Cochrane Central Register of Controlled Trials. We also searched for grey literature in Google Scholar. We complemented the initial search strategy by screening the reference lists from selected studies to identify any potentially relevant studies that may have been missed by searching clinical trial registries and contacting experts in the field to identify any unpublished or in-progress eligible studies (S1 Fig).

### Data management

All search results were uploaded to EndNote X8 for de-duplication. The resulting studies were uploaded to Distiller Systematic Review software for title/abstract and full-text screening. Results from the search strategy were documented per database before and after de-duplication.

### Study selection process

Study selection occurred in two phases (title/abstract and full-text screening). Two reviewers worked independently and in duplicate during each review phase to assess study eligibility. Chance-adjusted inter-rater agreement was assessed at each phase using the Kappa statistic (κ) [8]. A pilot test with a random sample of studies from the search strategy results was conducted before each screening phase to standardize the reviewers’ criterion. Disagreements were discussed, and the criterion was adapted if necessary. The pilot tests were repeated until a κ >0.70 was reached.

In the first phase, the title and abstract of all the studies obtained from the search strategy were screened, and reviewers selected the eligible articles based on the inclusion criteria. Discordant decisions passed to the full-text phase during this phase to achieve a highly sensitive selection. Eligibility was then assessed through full-text screening. Disagreements between reviewers during this phase were resolved by consensus, and if a consensus was not achieved, by a third reviewer’s arbitration. We documented the number of included and excluded articles and the reasons for the exclusion during the process.

### Data collection

Two independent reviewers working in duplicate collected data from all eligible articles using a web-based data extraction form. We gathered information regarding the type of study, title, author information, follow-up, year of publication, baseline characteristics of patients, primary/secondary outcomes, and interventions. Disagreements were resolved by consensus; if an agreement was not reached, a third reviewer made the final decision. Before this process, the two reviewers conducted a pilot test working independently and in duplicate. The reviewers provided feedback about any suggested adjustments and, if necessary, applied them to the preliminary extraction form.

### Missing data

If data important for our outcomes was missing or unclear, we contacted the corresponding author via e-mail to clarify the situation. After 10 days, a second e-mail was sent to non-responders. If there was no response, other authors were contacted. If, after all the attempts, no response was received, the study missing crucial data for the analysis was excluded. Every contact was documented.

### Risk of bias and quality assessment in individual studies

Two reviewers working independently and in duplicate evaluated the risk of bias using the Cochrane Risk of Bias tool for RCTs (RoB 2) [9]. The overall quality of the evidence of the primary outcomes was also assessed independently and in duplicate using the Grading of Recommendations Assessment, Development, and Evaluation (GRADE) system [10]. Any disagreement during this process was resolved by consensus or by a third reviewer’s arbitration.

### Subgroup & sensitivity analysis

A subgroup analysis was conducted based on the type of coronary disease and follow-up duration to explain inconsistencies between study results. Sensitivity analysis included studies comparing atorvastatin against placebo and studies with a low risk of bias if serious bias was observed that could impact the heterogeneity of the analysis for primary outcomes.

### Data synthesis

The studies were described as a narrative synthesis in a table, including study design and setting, sample size, target population characteristics, intervention description, study groups, and the risk of bias based on the RoB 2. We used R and RStudio with the (dmetar) and (meta) packages for statistical analysis [11–13]. The outcomes were summarized and presented as risk ratios (RR) with a 95% confidence interval (CI) for dichotomous outcomes and standardized mean differences (SMD) with a 95% CI for continuous outcomes. The Tau^2^ test and I^2^ statistic were used to assess heterogeneity between the studies. An I^2^ value >50% was considered indicative of considerable heterogeneity. A cumulative meta-analysis was performed when two or more studies were homogeneous enough. Since significant interstudy heterogeneity is expected in the report of clinical outcomes, a meta-analysis of these results using a random-effects model with a Mantel-Haenszel model without continuity correction was conducted. If this was not achievable, clinical outcomes were summarized narratively.

### Meta-bias

We assessed reporting bias of the RCT and quasi-RCT studies included by searching in ClinicalTrials.gov and evaluating if selective reporting of outcomes was present. Potential publication bias across the studies was assessed by funnel plots if more than 10 articles were included in the meta-analysis [14].

## Results

### Study characteristics & quality assessment

**Fig 1:**
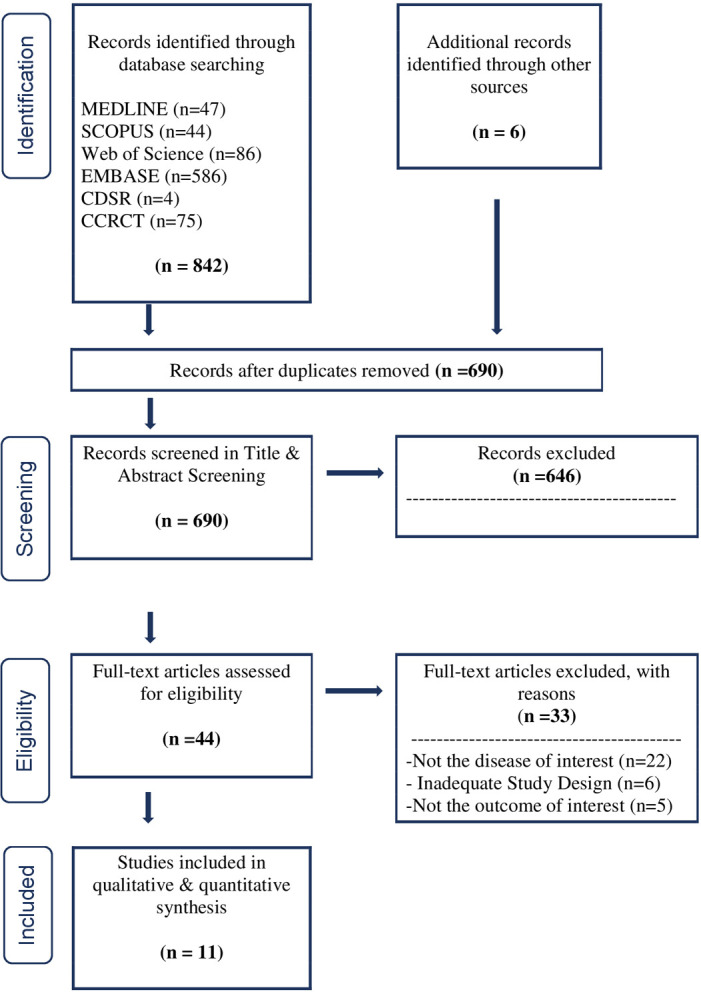
Study selection summary. CDSR: Cochrane Database of Systematic Reviews; CCRCT: Cochrane Central Register of Controlled Trials.

**Fig 2:**
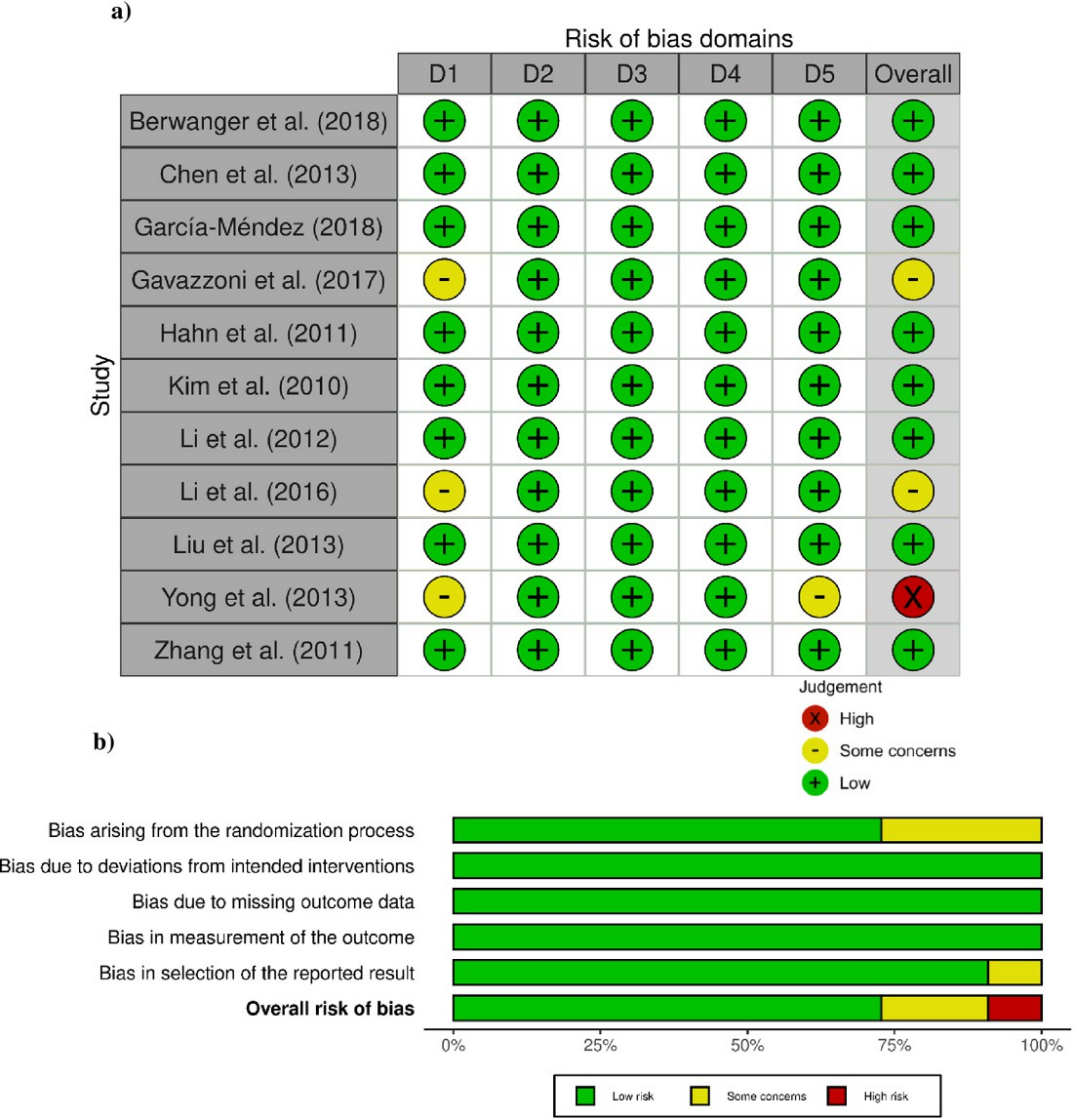
Risk of bias assessment & summary. D1: Bias arising from the randomization process; D2: Bias due to deviations from the intended intervention; D3: Bias due to missing outcome data; D4: Bias in the measurement of the outcome; D5: Bias in the selection of the reported result.

A total of (n = 842) studies were identified through the search strategy and (n = 6) through additional records encountered by the authors (Fig 1). After duplicate records were discarded (n = 690), records were screened based on Title & Abstract with a κ of 0.71, leaving (n = 44) records for full-text screening. Following the second phase screening (κ 0.88; n = 11), RCTs were included in the quantitative and qualitative analysis; all records included were classified with an overall low risk of bias except for n = 3 studies. Concerns arose due to bias in the randomization process and selection in the reported results (Fig 2). Reported funding for RCTs was by governmental agencies (n = 6) and (n = 1) private industry. The study period where patients were invited to participate ranged from 2007–17 in (n = 69) specialized centers or hospitals (Table 1). Additional information about outcomes and atorvastatin administration is found in S2 Table.

**Table 1:** Studies & patients characteristics.

Author	Country	Funding (Type)	Study Period	Multi-Center (Centers)	N Patients /males	N HDG/males	Mean Age (SD) HDG	N Control Group /males	Mean Age (SD) Control Group	Setting
**Kim et al. (2010)**	Korea	Yes (Government)	2007–08	Yes (4)	171/132	86/66	61 (±11)	85/66	59 (±11)	80 mg vs 10 mg
**Hahn et al. (2011)**	Korea	Yes (Government)	2007–09	Yes (5)	173/145	89/76	55.5 (±12.1)	84/69	59.7 (±12.8)	80 mg vs placebo
**Li et al.(2012)**	China	NR	2009–11	No	161/122	78/58	66.3 (±7.4)	83/64	65.4 (±7.2)	80 mg vs placebo
**Liu et al. (2013)**	China	Yes (Government)	2010–11	No	102/81	32/26	59.3 (±9.96)	70/55	62	80 mg vs placebo
**Gavazzoni et al. (2017)**	Italy	NR	2010	No	52/44	26/23	59 (±11)	26/21	57.8 (±13)	80 mg vs 20 mg
**Berwanger et al. (2018)**	Brazil	Yes (Government)	2012–17	Yes (53)	4191/3106	2087/1581	61.7 (±11.3)	2104/1525	61.9 (±11.7)	80 mg vs placebo
**García-Méndez et al. (2018)**	Mexico	Yes (Government)	2010–11	No	103/82	49/36	64 (±11)	54/46	64 (±11)	80 mg vs SC
**Li et al. (2018)**	China	NR	2012–13	No	118/81	59/42	63.17 (±10.2)	59/39	65.8 (±12.2)	80 mg vs 40 mg
**Zhang et al. (2011)**	China	Yes (Industry)	2009–10	No	112/94	52/42	62.17 (±10.8)	60/52	60.53 (±9.53)	80mg vs placebo
**Chen et al. (2013)**	China	NR	2007–10	No	156/109	76/55	60.71 (±12.4)	80/54	61.83 (±12.2)	80 mg vs placebo
**Yong et al. (2013)**	China	Yes (Government)	2010–11	No	60/49	20/18	54.5 (±12.7)	40/31	58.5	80 mg vs placebo

HDG: high-dose group; SD: standard deviation; SC: standard of care

### Patient characteristics

Information of (N = 5,399) patients was available; 2,477 presented NSTEMI, 2,654 were assigned to receive high-dose atorvastatin therapy (HDG), and 2,745 comprised the control group. Patients in the HDG had a mean age of 60.6 years and a proportion of 76.2% males compared to the control group, where the mean age was 61.4 years with 73.6 males. The comorbidities in each group were diabetes mellitus (31.8% vs. 30.7%), hypertension (68.0% vs. 68.7%), dyslipidemia (35.8% vs. 35.3%), chronic kidney disease (2.5% vs. 2.7%), and current smoker (33.7% vs. 36.0%). A relevant clinical history included previous MI (17.2% vs. 16.7%) and previous PCI (10.2 vs. 10.0%). The left ventricular ejection fraction had a mean of 51.8% in the HDG compared to 52.2% in the control group. Pre- and Post-procedural, in-hospital medication, and statin continuation characteristics after PCI are displayed in S3–S6 Tables.

### Clinical outcomes

The definition of MACE was assessed similarly across the included studies (S2 Table). For both groups of patients presenting with STEMI or NSTEMI, pre-loading a high dose of atorvastatin before PCI had an unclear effect in reducing the risk of MACE (RR: 0.56; 95% CI: 0.28–1.14; I^2^ = 62%; p = 0.091); contrasting with the sensitivity analysis (RR 0.78; 95% CI: 0.66–0.92; I^2^ = 0%; p = 0.0174). A sub-group analysis was performed to reduce heterogeneity; a similar unclear effect was observed in MACE in patients presenting with STEMI (RR: 0.51; 95% CI: 0.20–1.25; I^2^ = 67%; p = 0.104) and in MACE at 6 months of follow-up (RR: 0.52; 95% CI: 0.17–1.56; I^2^ = 0%; p = 0.124). These results were consistent with the sensitivity analysis for MACE in STEMI (RR 0.69; 95% CI: 0.42–1.12; I^2^ = 0%; p = 0.0816); sensitivity analysis on MACE at 6 months could not be performed due to the number of studies with low risk of bias (n = 1). In contrast, high-dose atorvastatin pre-loading showed a clear effect in reducing MACE at one month of follow-up (RR: 0.78; 95% CI: 0.67–0.91; I^2^ = 0%; p = 0.014) in both STEMI and NSTEMI. The number needed to treat (NNT) was 36 patients. This effect was also observed in the sensitivity analysis (RR 0.79; 95% CI: 0.63–0.98; I^2^ = 0%; p = 0.0429).

**Fig 3:**
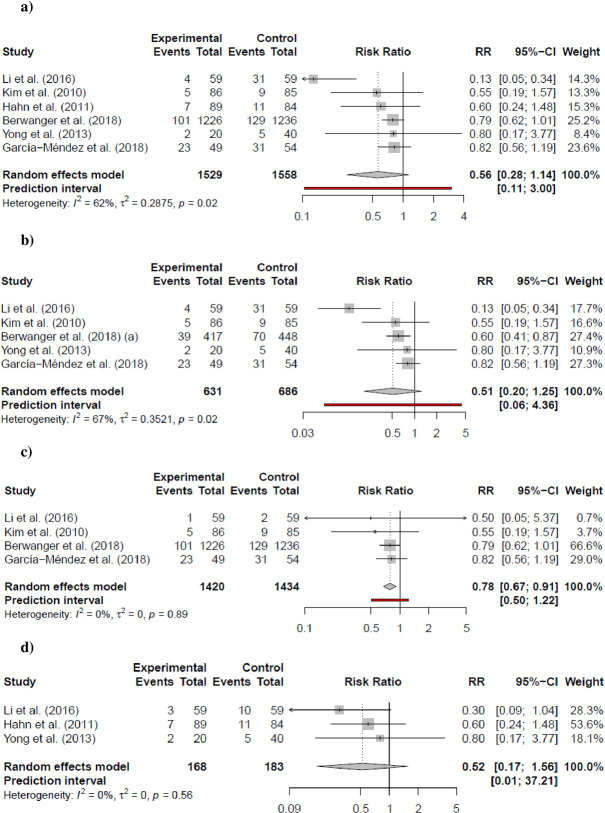
Primary outcomes. a) MACE b) MACE in STEMI c) MACE at 30-days d) MACE 6 months e) All-cause Mortality in STEMI. *p-value presented in the forests plots indicates the statistical significance of I^2^ tests.

**Fig 4:**
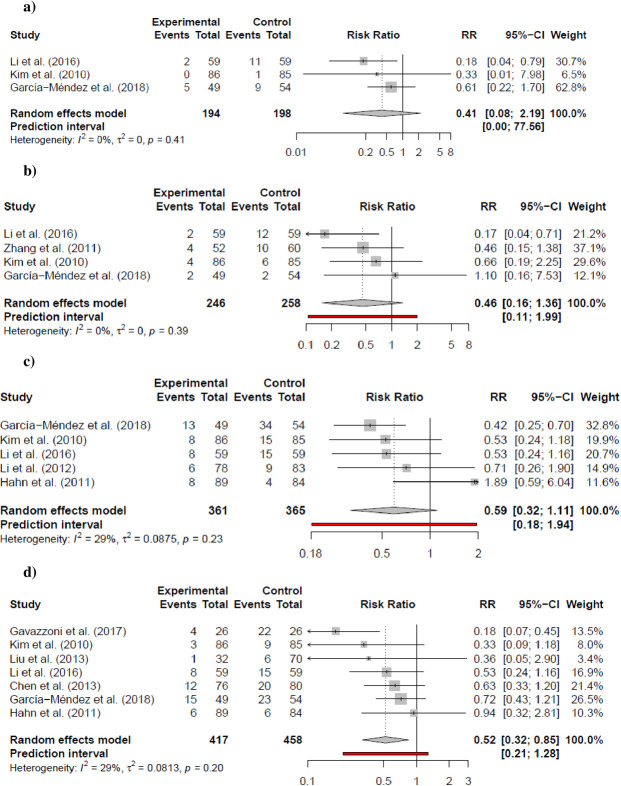
Secondary outcomes. a) Target Vessel Re-vascularization in STEMI b) Myocardial Infarction in STEMI c) Angiographic no-reflow in STEMI d) No TIMI Flow Grade III in STEMI e) Peak CK-MB in STEMI. *p-value presented in the forest plots indicates the statistical significance of I^2^ tests.

Data regarding all-cause mortality was found only in STEMI patients where a high dose of atorvastatin showed a clear effect on reducing the outcome (RR: 0.28; 95% CI: 0.10–0.81; I^2^ = 0%; p = 0.029) with an NNT of 14 patients; although the sensitivity analysis was not statistically significant (RR 0.33; 95% CI: 0.11–1.00; I^2^ = 0%; p = 0.0504). In the same manner, target vessel revascularization (RR: 0.41; 95% CI: 0.08–2.19; I^2^ = 0%; p = 0.147), myocardial infarction (RR: 0.46; 95% CI: 0.16–1.36; I^2^ = 0%; p = 0.106), and No TIMI flow III after PCI (RR: 0.52; 95% CI: 0.32–0.85; I^2^ = 29%; p = 0.017; NNT: 10) were only based on STEMI patients. No clear effect was observed in angiographic no-reflow (RR: 0.59; 95% CI: 0.32–1.11; I^2^ = 29%; p = 0.082) or mean differences in peak CK-MB (SMD: -0.16; 95% CI: -0.63–0.30; I^2^ = 76%; p = 0.383) (Figs 3 and 4). Sensitivity analyses were consistent with the main analysis (S2–S4 Figs). Certainty of the evidence is presented in Table 2.

**Table 2:** GRADE assessment summary for primary outcomes.

GRADE ASSESSMENT		Number of Patients		Effect		Certainty
Outcome	Number of Studies	Atorvastatin	Placebo	Relative Risk (95% CI)	Absolute Risk	
**MACE**	6	142/1529 (9.3%)	216/1558 (13.9%)	RR 0.56 (0.28 to 1.14)	6 fewer per 100 (from 10 fewer to 2 more)	⨁⨁◯◯ Low (due to inconsistency and imprecision)
**MACE at 30-days**	4	130/1420 (9.2%)	171/1434 (11.9%)	RR 0.78 (0.67 to 0.91)	3 fewer per 100 (from 4 fewer to 1 fewer)	⨁⨁⨁◯ Moderate (due to imprecision)
**All-cause Mortality in STEMI**	5	6/335 (1.8%)	30/342 (8.8%)	RR 0.28 (0.10 to 0.81)	6 fewer per 100 (from 8 fewer to 2 fewer)	⨁⨁⨁◯ Moderate (due to imprecision)

MACE: major adverse cardiovascular events; STEMI: ST-elevated myocardial infarction

## Discussion

### Summary of findings

Information obtained from (n = 5,399) STEMI and NSTEMI patients included in (n = 11) studies generated a clinical landscape for clinicians and patients about the effects of high-dose atorvastatin therapy before performing PCI [15–24]. Our findings suggest that pre-loading with high-dose atorvastatin reduces the incidence of MACE at 30 days of follow-up and all-cause mortality in STEMI patients, with low and moderate certainty, respectively. A reduction in the number of patients not reaching TIMI Flow Grade III was observed in the atorvastatin group. No clear effect was observed in patient-important outcomes such as MI, MACE at 6 months of follow-up, target vessel revascularization, angiographic no-reflow, or peak CK-MB in STEMI patients. These findings suggest that high-intensity atorvastatin therapy confers benefits in important outcomes such as MACE at 30 days and all-cause mortality in STEMI patients. Our results emerged from RCTs with an overall low risk of bias, except in three, where possible bias derived mainly from the allocation-concealment process. In this clinical scenario, it could be debatable if this potentially affects the measured outcome. The study rated with a high risk of bias raised concerns due to the study including only patients with a final TIMI flow grade > II, which omitted a relevant group of patients [24].

### Strengths & limitations

Our robust methodological approach in study selection provided a group of patients that could represent a real-world scenario, especially for STEMI patients. Even though the p-value of some patient-important and angiographic outcomes could be considered non-significant, this result could be due to the total number of patients included in the RCTs, limiting the possibility of performing a valid time-to-event effect estimate. Despite this limitation, our narrow prediction intervals support a strong possibility for atorvastatin to have a clear beneficial effect across various outcomes in the short term (30 days). On the other hand, evidence of NSTEMI on the effects of all-cause mortality is limited. Similarly, our results are limited in patients presenting STEMI or NSTEMI with a left main coronary artery lesion.

### Comparison with other studies

Ma et al. explored MACE at 30 days in STEMI and NSTE-ACS, only to find a clear effect in STEMI patients [25]. This contrasts with our results, where we observed a clear benefit in STEMI and NSTEMI, which could have arisen from excluding unstable angina patients. On the other hand, we demonstrated a clear effect of atorvastatin in reducing all-cause mortality in STEMI patients. Ma et al. could not demonstrate such an effect in ACS patients. Borovac et al. explored myocardial infarction and all-cause mortality in ACS patients, contrasting only in all-cause mortality [26]. Liu et al. explored clinical and procedural outcomes similar to our review [27]. Atorvastatin showed no clear effect in reducing the mean peak CK-MB of ACS patients, in accordance with our results in STEMI patients. The angiographic outcome of TIMI flow grade III after PCI showed, in both reviews, a favorable effect in the atorvastatin group.

### Implications for future research

High-dose pre-loading with atorvastatin before PCI in STEMI and NSTEMI patients seems to be a great secondary prevention strategy in the 30 days following the intervention. The effects of atorvastatin were observed in patients with various co-morbidities, independently of their statin naïve status. Future research should focus on the short-term outcomes since we demonstrated that these effects are not observed at 6 months of follow-up. Evidence on the implementation strategies of pre-loading with atorvastatin, such as timing (early <12h vs. late >12) and route of administration, are needed.

## Conclusion

Many factors play a role in the prognosis of patients after PCI, especially for STEMI and NSTEMI. In the era of patient-centered medicine, individualization of therapeutic strategies is needed based on cardiovascular risk and comorbidities. Patients presenting with STEMI or NSTEMI benefit from high-dose atorvastatin pre-loading before PCI, reducing MACE at 30 days. The use of high-dose atorvastatin in STEMI patients reduced all-cause mortality. The beneficial effects of atorvastatin pre-loading are limited to 30 days post-PCI. Future research should focus on the timing of administrating high dose atorvastatin.

## Supporting information

S1 Fig Search strategy. (TIFF)

S2 Fig Sensitivity analysis of primary outcomes. (TIFF)

S3 Fig Sensitivity analysis of secondary outcomes. (TIFF)

S4 Fig Sensitivity analysis atorvastatin vs placebo. (TIFF)

S1 Table PRISMA checklist. (DOCX)

S2 Table Outcomes and atorvastatin administration characteristics. (TIFF)

S3 Table Patients’ relevant co-morbidities. (TIFF)

S4 Table Patients’ infarction characteristics. (TIFF)

S5 Table Patients’ in-hospital medication. (TIFF)

S6 Table Patients’ post-percutaneous coronary intervention characteristics. (TIFF)
