# Supplementary figures and images for: Atorvastatin before percutaneous coronary intervention: A systematic review and meta-analysis
Source: PLoS One. 2024 Jan 2;19(1):e0293404. doi: 10.1371/journal.pone.0293404 (PMC10760670; doi:10.1371/journal.pone.0293404)

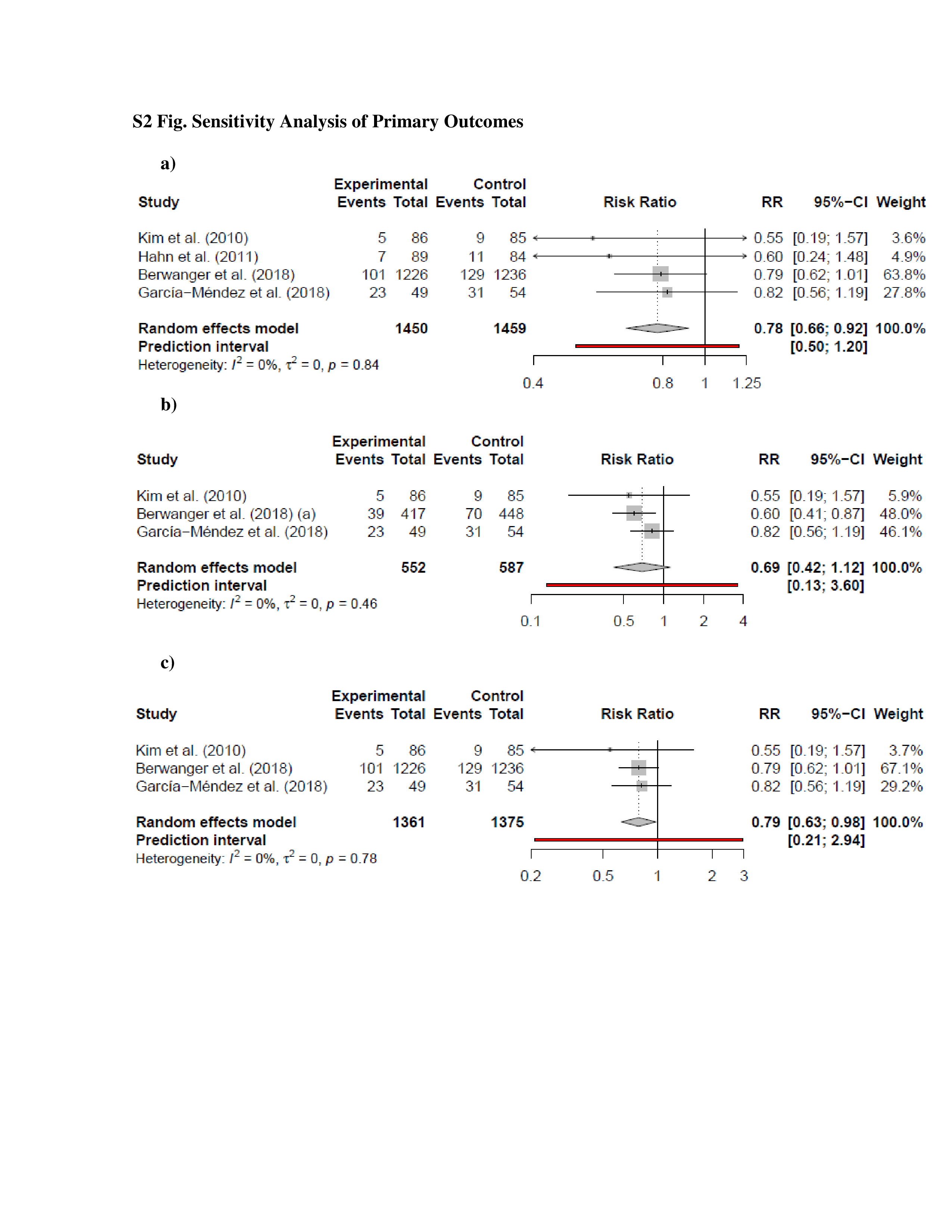

Supplement: S2 Fig — (TIFF) [file pone.0293404.s002.tiff]

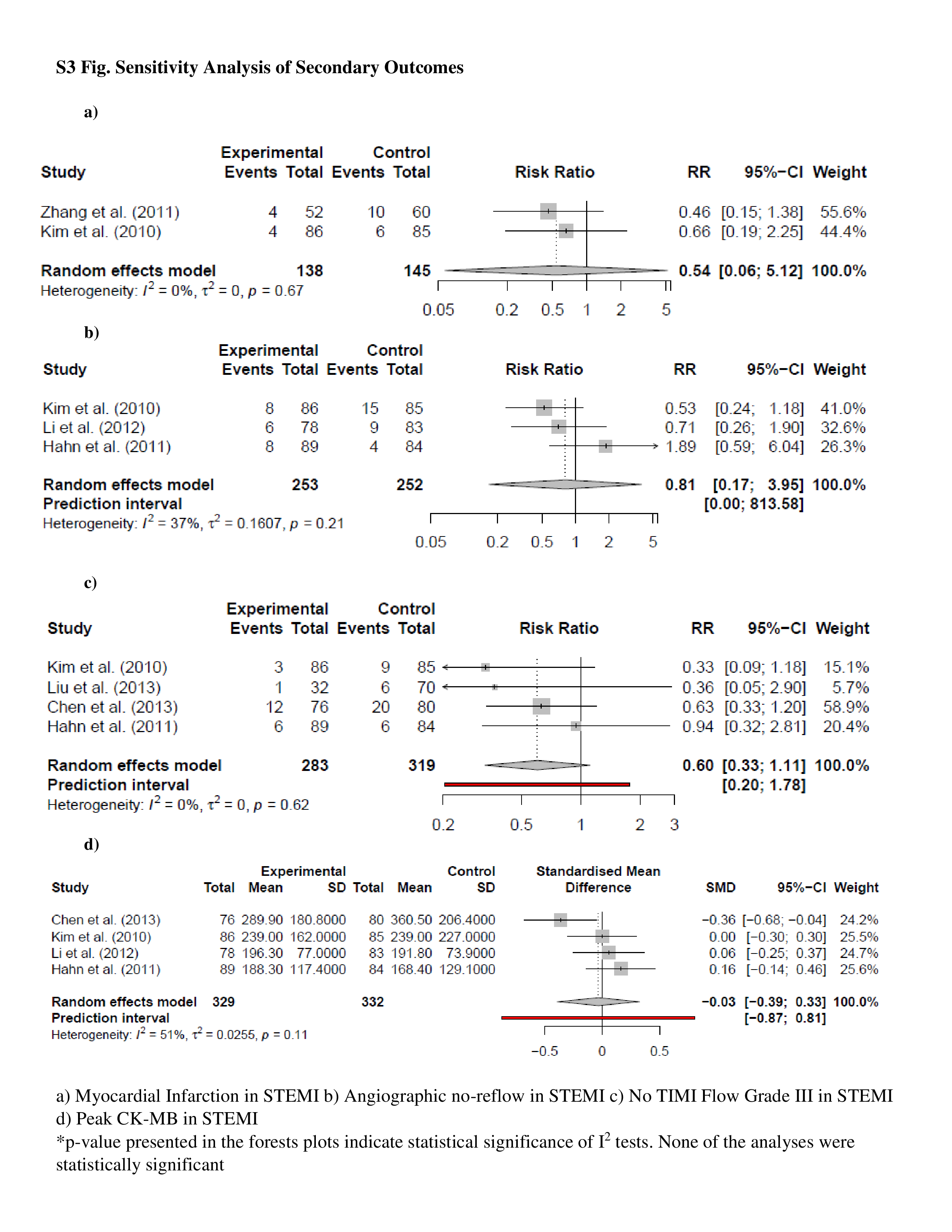

Supplement: S3 Fig — (TIFF) [file pone.0293404.s003.tiff]

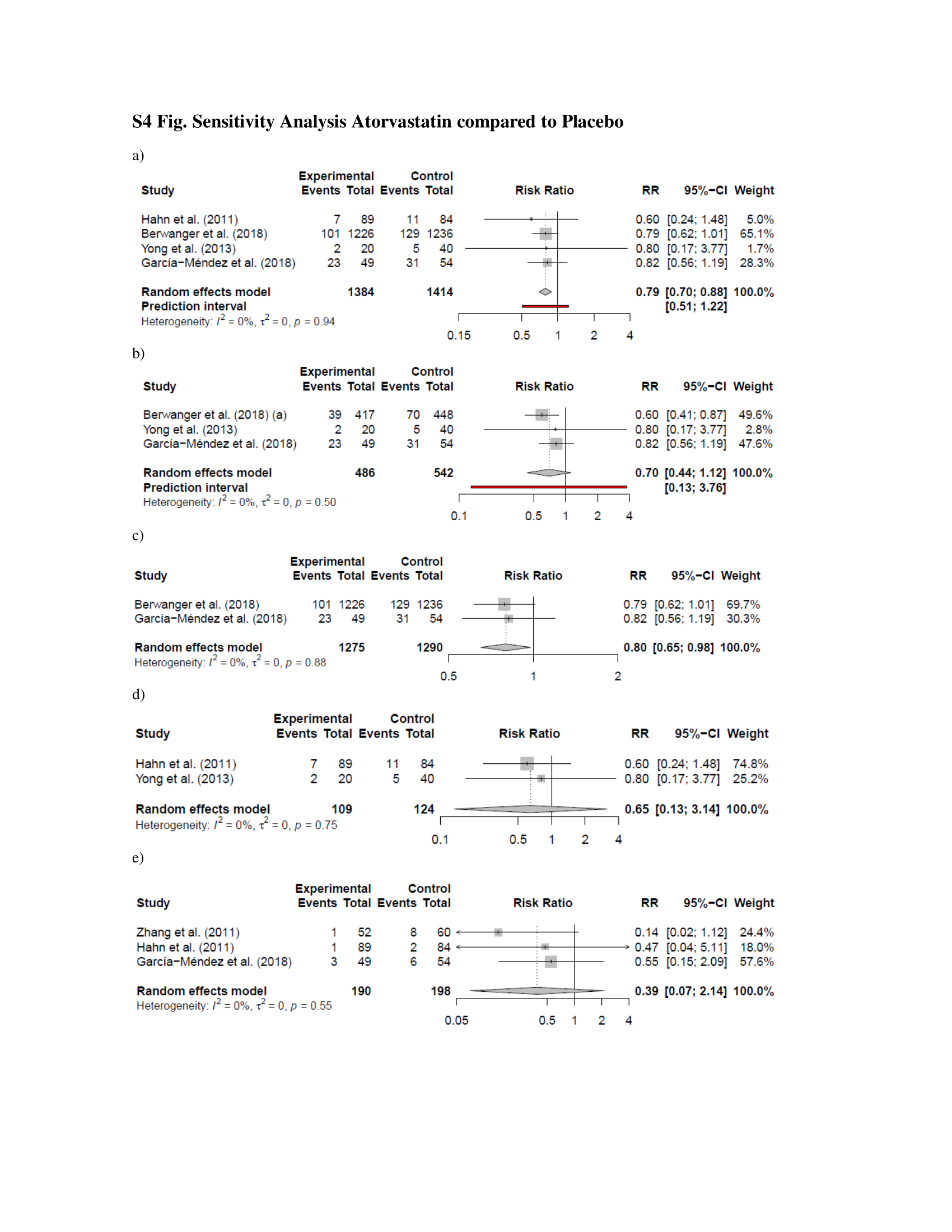

Supplement: S4 Fig — (TIFF) [file pone.0293404.s004.tiff]
